# The Efficacy of S-Adenosyl Methionine and Probiotic Supplementation on Depression: A Synergistic Approach

**DOI:** 10.3390/nu14132751

**Published:** 2022-07-01

**Authors:** Hammad Ullah, Ayesha Khan, Kannan R. R. Rengasamy, Alessandro Di Minno, Roberto Sacchi, Maria Daglia

**Affiliations:** 1 Department of Pharmacy, University of Napoli Federico II, Via D. Montesano 49, 80131 Naples, Italy; hammad.ullah@unina.it (H.U.); alessandro.diminno@unina.it (A.D.M.); 2 Department of Medicine, Combined Military Hospital Nowshera, Nowshera 24110, Pakistan; ayeshakhan13012@gmail.com; 3 Centre for Transdisciplinary Research, Department of Pharmacology, Saveetha Dental College, Saveetha Institute of Medical and Technical Sciences (SIMATS), Chennai 600077, India; rengasamy@iceir.net; 4 CEINGE-Biotecnologie Avanzate, Via Gaetano Salvatore 486, 80145 Naples, Italy; 5 Applied Statistic Unit, Department of Earth and Environmental Sciences, University of Pavia, Viale Taramelli 24, 27100 Pavia, Italy; roberto.sacchi@unipv.it; 6 International Research Center for Food Nutrition and Safety, Jiangsu University, Zhenjiang 212013, China

**Keywords:** depression, S-adenosyl methionine, probiotics, gut dysbiosis, synergistic effects

## Abstract

Depression is a common and serious health issue affecting around 280 million people around the world. Suicidal ideation more frequently occurs in people with moderate to severe depression. Psychotherapy and pharmacological drugs are the mainstay of available treatment options for depressive disorders. However, pharmacological options do not offer complete cure, especially in moderate to severe depression, and are often seen with a range of adverse events. S-adenosyl methionine (SAMe) supplementation has been widely studied, and an impressive collection of literature published over the last few decades suggests its antidepressant efficacy. Probiotics have gained significant attention due to their wide array of clinical uses, and multiple studies have explored the link between probiotic species and mood disorders. Gut dysbiosis is one of the risk factors in depression by inducing systemic inflammation accompanied by an imbalance in neurotransmitter production. Thus, concomitant administration of probiotics may be an effective treatment strategy in patients with depressed mood, particularly in resistant cases, as these can aid in dysbiosis, possibly resulting in the attenuation of systemic inflammatory processes and the improvement of the therapeutic efficacy of SAMe. The current review highlights the therapeutic roles of SAMe and probiotics in depression, their mechanistic targets, and their possible synergistic effects and may help in the development of food supplements consisting of a combination of SAMe and probiotics with new dosage forms that may improve their bioavailability.

## 1. Introduction

Depression is a common and serious mood disorder worldwide, and subjects with depression often experience persistent feelings of sadness and hopelessness and loss of interest in daily life activities. The American Psychiatric Association’s Diagnostic and Statistical Manual of Mental Disorders, Fifth Edition (DSM-5), categorizes depression into disruptive mood dysregulation disorder, major depressive disorder (MDD), premenstrual dysphoric disorder, dysthymia (persistent depressive disorder), and depressive disorder due to another medical condition [1]. According to World Health Organization (WHO) estimates, approximately 280 million people are living with depression around the globe [2]. When recurrent, depression may become a moderate to serious health issue. Suicidal ideation is more common in depressed individuals, especially in MDDD or other depressive disorders with moderate to severe intensity, and over 700,000 people die each year due to suicidal attempts [2].

Subthreshold depression (SD), also known as subsyndromal or subclinical depression, is defined by the presence of two or more depressive symptoms for 2 weeks, while not meeting the criteria for a diagnosis of major depressive disorder (MDD) and/or dysthymia, although it may possess a significant impact on the quality of an individual’s life [3]. The prevalence of SD varies highly across scientific studies, as prevalence rates for SD range from 2.9% to 9.9% in primary care and 1.4% to 17.2% in community settings [4]. SD is a risk factor in the development of MDD, especially in the older population, although literature data suggest that it may not be merely a prodromal phase of MDD, and it may not always lead to MDD [5].

Both SD and MDD are associated with increased mortality, possibly due to risky behavior and physical comorbidities [6]. The aim of treatment is to reduce or prevent the negative outcomes associated with depressive conditions [7]. Psychotherapy and the use of psychotropic agents for short periods have been recommended as first-line therapeutic options, though pharmacological treatment has been associated with numerous adverse drug reactions (ADRs) [7]. Available pharmacological classes employed in the treatment of depressive disorders are tricyclic antidepressants (TCAs), selective serotonin reuptake inhibitors (SSRIs), serotonin and norepinephrine reuptake inhibitors (SNRIs), and monoamine oxidase inhibitors (MAOIs) [8]. Targeting and treating SD and mild to moderate depression, particularly in the high-risk population, might be a useful strategy in decreasing the prevalence rates of MDD [9]. A study conducted by Williams et al. (2000) showed the improvement of depressive symptoms and the positive impact of antidepressants in older patients with minor depression and functional impairment [10]. By contrast, a meta-analysis report including six clinical trials showed no significant advantage of antidepressants in the treatment of SD over placebo [11].

About 30% of patients do not respond well to the recommended doses of antidepressant medications, and this is termed as refractory depression. In such cases, increasing the dosage of the existing antidepressant, switching between different pharmacological classes, and/or adding lithium therapy has been suggested, though none of these approaches thoroughly resolves the issue [12]. Conventional antidepressant therapies can provoke tolerability or acceptability issues in patients [13,14]. The most notable adverse drug reactions (ADRs) reported after treatment with antidepressant drugs include dry mouth, sexual dysfunction, dizziness, hypotension, appetite changes, and insomnia [13]. Discontinuation syndrome, characterized by flulike symptoms, headache, dizziness, shocklike sensations in the upper extremities, vivid dreams, and impaired concentration are also common with abrupt discontinuation of antidepressants [15]. A meta-analysis of randomized controlled trials (RCTs) to evaluate the efficacy and acceptability of antidepressant medications among patients with generalized anxiety disorders (GADs) showed higher dropout rates for patients with older antidepressants, such as TCAs excluding imipramine, as compared with newer antidepressants (i.e., venlafaxine and paroxetine), showing poor acceptability of the older antidepressants by patients, which may be correlated with the intolerable side effects of this class of drugs [14].

Recent literature has linked poor diet and lack of exercise with the genesis and course of depression. Thus, recommendations regarding dietary improvement in combination with an increase in exercise and cessation of smoking should be routinely given to patients with depression [16]. Dietary improvement and supplementation could be an acceptable and less expensive strategy to be used alternatively in mild cases or in combination with standard antidepressant therapy in cases of moderate to severe depression [17]. A systemic review studied the association between diet and depression in children and adolescents, which led to the conclusion that a high intake of healthy foods (fruits, leafy green vegetables, and fish consumption) and a lower intake of unhealthy foods (fast foods, snacking, and confectionery/sweets) are reciprocal to low mood and depressive episodes. Moreover, no significant association of atypical mood was found with cereal/grain, dairy product, and fat consumption [18]. Individual bioactive components showing an improvement of depression and anxiety symptoms include vitamin D, antioxidant vitamins (A, C, and E), polyunsaturated fatty acids (PUFAs) (eicosapentaenoic acid and docosahexaenoic acid) [19], N-acetylcysteine (NAC) [20], S-adenosyl methionine (SAMe) [21], prebiotics and probiotics [22], and essential trace elements (selenium, zinc, magnesium, copper, iron, and chromium) [23]. This review is designed to focus and highlight the antidepressant potential of SAMe and probiotic strains, their mechanistic targets and synergistic effects, the possible new formulations for SAMe to improve its bioavailability, and the possibility of the development of novel food supplements containing both SAMe and probiotic strains.

## 2. SAMe and Neurobiological Pathways Related to Depression

**Figure 1:**
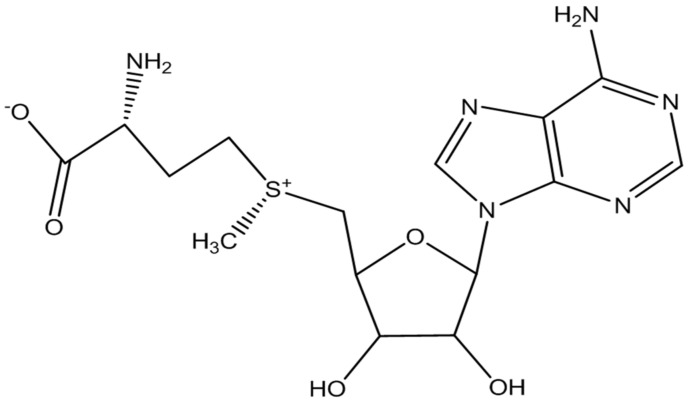
Chemical structure of SAMe molecule.

SAMe (Figure 1) was discovered in 1952 by the Italian scientist Giulio Cantoni [24]. It is an endogenous molecule occurring naturally in the human body that regulates a plethora of biochemical pathways, including the synthesis and metabolism of neurotransmitters and potential epigenetic pathways [25]. The biosynthesis of SAMe is carried out primarily in the liver by the conversion of L-methionine into SAMe [26]. Transmethylation, trans-sulfuration, and aliphatic polyamine synthesis are the major roles of SAMe in cell function and metabolism, influencing SAMe availability, which in turn can result in abnormalities in cell function and metabolism [27]. SAMe concentration can be measured in the blood and cerebrospinal fluid (CSF), with ranges established for normal and disease states. Low CSF levels of SAMe have been reported in cases of inherited defects in folate and methionine metabolism, depressive disorders, Alzheimer’s disease, Parkinson’s disease, and HIV infections [28]. Folate and vitamin B12 deficiencies may account for the low SAMe levels, particularly in individuals with depression and dementia, as both are necessary cofactors in the synthesis of SAMe [29].

### 2.1. Antidepressant Efficacy of SAMe

Routinely prescribed in Europe for decades and gaining popularity in the United States as a dietary supplement since 1998–1999, SAMe has been proposed for many medical conditions, particularly depressive disorders [30]. SAMe has been widely studied compared with other food supplement ingredients, and an impressive body of literature from the last few decades suggests its antidepressant efficacy [31]. SAMe can cross the blood–brain barrier (BBB) from an oral dose, resulting in increased CSF levels, including in patients with psychiatric disorders [28]. As evidenced from available literature data, oral SAMe in the dose range of 200–1600 mg/day is superior to placebo and is as effective as TCAs in alleviating different types of depression. Furthermore, SAMe has been reported to have a faster onset of action than conventional antidepressant agents, and it may potentiate the therapeutic response of these drugs [30]. Table 1 summarizes the clinical trials, illustrating the effects of SAMe in depressive disorders.

Sarris et al. (2020) tested the efficacy of SAMe versus placebo in MDD-diagnosed patients with mild to moderate depressive symptoms in a double-blind randomized controlled trial (RCT) [32]. A total of 49 patients undergoing no antidepressant therapy were treated with SAMe monotherapy (800 mg/day) or placebo for 8 weeks. The relationships between therapeutic response and once-carbon cycle biomarkers, brain-derived neurotrophic factor (BDNF), and relevant single-nucleotide polymorphisms (SNPs) were analyzed. The results showed a modest but statistically insignificant improvement of depressive symptoms in both SAMe and placebo groups. Increased folate concentrations were correlated with improved symptoms in the SAMe-treated group. Moreover, no change in one-carbon biomarkers, BDNF, and SNPs was observed in relation to the therapeutic response.

The role of betaine in improving the antidepressant effects of SAMe was evaluated in patients with mild to moderate depression in a randomized, observational, controlled trial [33]. A total of 46 subjects were enrolled in the study and were treated with SAMe (800 mg/day) or a combination of SAMe (750 mg/day) and betaine (375 mg/day) for 90 days. Both treatments showed similar results in the improvement of symptoms, such as feelings of hopelessness, worthlessness, anxiety, psychomotor agitation, physical efficiency, and somatization, but a combination of SAMe and betaine demonstrated better, statistically significant, results at the end of the therapy period.

SAMe-induced increases in the effects of serotonin reuptake inhibitors (SRIs) on the cognitive symptoms of MDD were studied in a 6-week double-blind randomized clinical trial [34]. A total of 46 patients (aged 18–80) with MDD and nonresponsive to SRIs were recruited in the study and treated with placebo or SAMe pills (800 mg/day), an adjunct to SRIs at adequate doses (20 mg/day or more for fluoxetine, citalopram, or paroxetine; 10 mg/day or more for escitalopram; 50 mg/day or more for sertraline; 60 mg/day or more for duloxetine; and 150 mg/day or more for venlafaxine). A significant improvement of the ability of patients to recall information was observed with adjunctive SAMe compared with placebo, suggesting that SAMe may improve memory-related cognitive symptoms in depressed patients.

Mischoulon et al. (2013) performed a double-blind randomized placebo-controlled clinical trial to demonstrate a comparative therapeutic response of SAMe and escitalopram in MDD [35]. A total of 189 patients (mean age: 45 years) were treated with SAMe (1600–3200 mg/day), escitalopram (10–20 mg/day), or placebo for 12 weeks. Clinical response was measured using the 17-item Hamilton Depression Rating Scale (HDRS-17), and doses were increased at 6 weeks in nonresponders. No significant differences were observed in patients treated with SAMe or escitalopram. The remission rates for SAMe and escitalopram were 28% and 17% for the placebo group. Tolerability was evaluated by the Systematic Assessment for Treatment of Emergent Events-Specific Inquiry (SAFTEE-SI), which showed good tolerability in the SAMe arm, with few and mild gastrointestinal (GI) side effects being noted.

In another study, Sarris et al. (2014) examined the comparative therapeutic response of SAMe and escitalopram in MDD, which showed superior efficacy of SAMe in comparison with escitalopram in the treatment of depressive disorders [36]. A total of 144 patients were recruited in the randomized clinical trial and were treated with SAMe (1600 mg/day), escitalopram (10 mg/day), or placebo for 12 weeks. Regarding the SAMe and escitalopram groups, the subjects who did not respond to treatment after 6 weeks were treated with SAMe and escitalopram at doses of 3200 and 20 mg/day, respectively. On the primary outcome of HAMD-17, a significant improvement of the depressive symptoms was seen in the SAMe and escitalopram groups. The efficacy of SAMe was superior to that of escitalopram, as the remission rates reported were 34% for SAMe, 23% for escitalopram, and 6% for placebo.

Augmentation of SRIs after SAMe intake was evaluated by Papakostas et al. (2010) in nonresponder patients with MDD in a double-blind randomized clinical trial [37]. A total of 73 SRI nonresponders aged 18–80 years were recruited for the clinical trial and were treated with adjunctive oral SAMe with a target dose of 1600 mg/day or placebo for 6 weeks. The clinical response was assessed by HDRS-17, which showed a better responsive and remission rate with adjunctive SAMe (36.1% and 25.8%), compared with placebo (17.6% and 11. 7%). SAMe supplementation demonstrated a significant improvement of depressive symptoms for parkinsonian patients in a pilot study [37]. Patients with Parkinson’s disease and concurrent depression were treated with SAMe (800–3600 mg/day) for 10 weeks, where 11 patients completed the study, and 2 patients prematurely terminated the cycle because of increased anxiety. A total of 10 patients reported an improvement of at least 50% on the HDRS-17 scale, while 1 patient did not show improvement of the depressive symptoms.

Sexual dysfunction is one of the most commonly reported ADRs associated with antidepressant drugs, and this may potentially affect adherence to the therapy. Thus, it is more vital to develop novel treatments that target antidepressant-induced sexual dysfunction and/or can be used safely in mild to moderate depression without such unfavorable adverse events [38]. Dording et al. (2012) examined the potential of adjunctive SAMe to improve sexual dysfunction over a 6-week monocentric randomized double-blind clinical trial [39]. A total of 73 patients (age: 18–80 years) were randomized to receive SAMe (800 mg/day) or placebo augmentation of SSRI/SNRI, and sexual functioning was measured using the Massachusetts General Hospital–Sexual Functioning Questionnaire (MGH-SFQ). The results showed significantly lower arousal and erectile dysfunction at endpoint in men treated with adjunctive SAMe than those treated with placebo.

However, based on available literature, a final conclusion cannot be drawn, and larger randomized double-blind controlled studies are necessary to confirm the clinical efficacy of SAMe against depressive disorders using antidepressants from different classes, as most of the current studies compare the efficacy of SAMe with placebo, but it is also obvious to observe and compare the efficacy of SAMe with antidepressant drugs from each class. Safety should also be one of the main concerns and will be assessed in detail, in particular the induction of mania [40,41].

**Table 1:** Efficacy of SAMe in depression, as evidenced from clinical trials.

Mental Condition	Study Design	Intervention	Main Findings	References
MDD, with mild to moderate depression symptoms	Double-blind RCT. A total of 49 patients with no concurrent antidepressant therapy were recruited in the trial.	SAMe (800 mg) or placebo for 8 weeks.	Modest improvement of depressive symptoms in both SAMe and placebo groups. Increased folate concentrations in SAMe treated patients, correlated with improvement in symptoms. No change in one-carbon cycle biomarkers, BDNF, and SNPs.	[32]
Mild to moderate depression	Randomized observational controlled trial. A total of 46 subjects were enrolled in the trial.	SAMe (800 mg/day) (*n* = 23) or SAMe (750 mg/day + betaine 375 mg/day) (*n* = 23) for 90 days.	Improvement of depressive symptoms in both groups. Combination of SAMe and betaine demonstrated more effectiveness in the remission of symptoms, with mild to moderate depression.	[33]
MDD and cognitive deficits	Double-blind RCT. A total of 46 SRI nonresponders with MDD were selected.	Adjunctive SAMe (800 mg/day) or placebo for 6 weeks.	Significant improvement in memory-related cognitive symptoms.	[34]
MDD	Double-blind RCT. A total of 189 patients were recruited in the study for comparative analysis of SAMe and escitalopram.	SAMe (1600–3200 mg/day), escitalopram (10–20 mg/day), or placebo for 12 weeks.	No significant differences were noted in remission rates with SAMe and escitalopram. SAMe was more tolerable, with mild to moderate GI side effects.	[35]
MDD	Double-blind RCT. A total of 144 patients were recruited in the study for comparative analysis of SAMe and escitalopram.	SAMe (1600–3200 mg/day), escitalopram (10–20 mg/day), or placebo for 12 weeks.	SAMe was slightly more efficacious than escitalopram. The remission rates were 34% for SAMe, 23% for escitalopram, and 6% for placebo.	[36]
MDD	Double-blind RCT. A total of 73 SRI nonresponders (age: 18–80 years) were selected for the study.	Adjunctive oral SAMe with a target dose of 1600 mg/day or placebo for 6 weeks.	Responsive and remission rates with adjunctive SAMe were 36.1% and 25.8%, respectively, as compared with placebo (17.6% and 11. 7%).	[42]
Depression in patients with PD	Open-label clinical trial. A total of 13 patients were enrolled in the study.	SAMe (800–3600 mg/day).	A total of 11 patients completed the study, and 2 prematurely terminated because of increased anxiety. A total of 10 patients demonstrated at least 50% improvement on the HDRS-17 scale.	[37]
Antidepressants induced sexual dysfunction	Double-blind RCT. A total of 73 patients (age: 18–80 years) were randomized in two treatment groups (SAMe or placebo).	Augmentation of SSRI/SNRI with SAMe (800 mg/day) or placebo for 6 weeks.	Significant improvement in arousal and erectile dysfunctions in SAMe-augmented men at endpoint as compared with placebo-treated patients.	[39]

Major depressive disorders (MDDs), randomized clinical trial (RCT), S-adenosyl methionine (SAMe), brain-derived neurotrophic factor (BDNF), single-nucleotide polymorphisms (SNPs), serotonin reuptake inhibitors (SRIs), selective serotonin reuptake inhibitors (SSRIs), serotonin and norepinephrine reuptake inhibitors (SNRIs), 17-item Hamilton Depression Rating Scale (HDRS-17).

### 2.2. Mechanisms of Action

#### 2.2.1. Transmethylation Pathways

SAMe is one of the major methyl donors, exerting its effects on the central nervous system (CNS) via cellular transmethylation pathways. The methylation of several catecholamine moieties present in neurotransmitters, epigenetic targets (DNA, RNA, and histones), and protein phosphatase 2A is the cellular target of SAMe transmethylation pathways pertinent to mental and neurological diseases [43]. The regulation of DNA and RNA methylation by SAMe helps in controlling transcription processes. Messenger RNA (mRNA) hypomethylation, low transcription rates, and disrupted splicing patterns are events commonly occurring in SAMe depletion states. Hypomethylation of ribosomal RNA (rRNA) in the nucleus may lead to the inhibition of its cytoplasmic export, and ultimately results in the further inhibition of mRNA processing [44]. A review on DNA methylation in depression demonstrated a connection between depression and methylation of BDNF and NR3C1 genes, while the correlation of SLC6A4 with depression was conflicted [45].

Arginine methylation of RNA binding proteins in arginine flanked by glycine (RGG) domains may alter the processing of mRNA associated with specific RNA binding proteins, where protein arginine methyltransferase (PRMT), whose activity is SAMe level dependent, catalyzes this type of methylation [46]. The involvement of SAMe in the methylation of histone residues can cause both the activation and repression of genes, depending on the lysine residue involved [47]. Evidence also exists that supports SAMe-mediated anti-inflammatory effects by decreasing the expression of proinflammatory cytokines, such as tumor necrosis factor alpha (TNF-α) via DNA and histone methylation. This could be more important in the treatment of depression, as inflammation is one of the contributors to the initiation and progression of this disease [48,49].

Interestingly, several conventional psychiatric drugs have been found to modify epigenetic pathways. For instance, antidepressants (amitriptyline and escitalopram) and mood stabilizers (valproate) may inhibit DNA methylation through the downregulation of DNA methyltransferases (DNMTs), while fluoxetine (SSRI) can inhibit histone methylation [47]. This suggests that adjunct administration of SAMe supplements might influence the conventional pharmacotherapy of psychiatric diseases through common epigenetic pathways [50].

Phospholipids play a vital role in the composition of the cell membrane and membrane dynamic functions, which are significant for the maintenance of healthy neurons and adequate neurotransmission, thus preventing depression [29]. SAMe may enhance the fluidity of the cell membrane by methylation of membrane-bound phosphatidylethanolamine, altering the organization of lipid microdomains and thus modulating the function of several membrane-bound monoamine receptors and monoamine transporters [31].

#### 2.2.2. Monoamine Neurotransmitters

**Figure 2:**
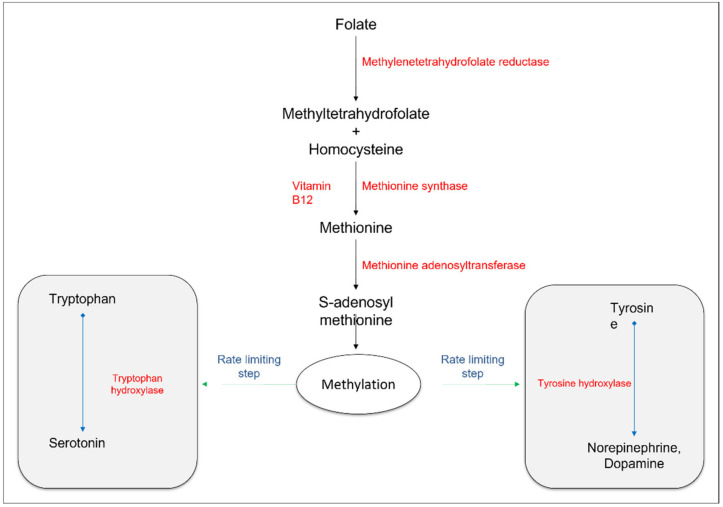
Biochemical synthesis of SAMe and its potential role in the synthesis of neurotransmitters. SAMe is synthesized in the body through a series of biochemical reactions involving folate, vitamin B12, and certain enzymes. SAMe acts as a methyl-donating cofactor in the rate-limiting steps of the chemical reactions, leading to the synthesis of serotonin, norepinephrine, and dopamine.

The link between SAMe and its associated mood-enhancing effects might also be explained by its potential role in the synthesis of monoamine neurotransmitters, such as norepinephrine, dopamine, and serotonin (Figure 2) [51]. Norepinephrine and dopamine are known to synthesize from tyrosine in a series of chemical reactions, dependent on the enzyme tyrosine hydroxylase [52]. Similarly, serotonin is synthesized from tryptophan in a series of chemical reactions, the rate-limiting step of which is dependent on the enzyme tryptophan hydroxylase [53]. SAMe acts as a methyl-donating cofactor in the rate-limiting steps of these chemical reactions to enhance the levels of neurotransmitters [54]. In addition, the folate cycle is an essential mechanistic pathway for the synthesis and regulation of tetrahydrobiopterin, an important cofactor for the enzymes responsible for the conversion of amino acids to monoamine neurotransmitters [54,55]. Bottiglieri et al. investigated the effects of SAMe on neurotransmitter levels and depressed mood. They found that low levels of SAMe and, consequently, low CSF levels of serotonin, norepinephrine, and dopamine metabolites were associated with high levels of homocysteine [56], which in turn is considered a possible factor for mood depression [57].

### 2.3. Safety Concerns

Oral intake of SAMe supplements appears to be nontoxic up to doses of 1600 mg/day [58]. Overall, SAMe has a favorable safety profile with no sexual and cognitive/memory dysfunction being reported. The most common SAMe ADRs include nausea and, less frequently, diarrhea, vomiting, and abdominal discomfort [28]. The induction of hypomanic or manic symptoms with SAMe supplementation in patients with bipolar disorders is a serious concern, and thus, it should be used cautiously in vulnerable individuals [59].

Targum et al. (2018) conducted a 6-week double-blind placebo-controlled augmentation study to compare the efficacy and safety of SAMe in combination with ongoing antidepressant treatment. A total of 234 subjects were recruited in the study and were supplemented with SAMe (800 mg) (*n* = 118) or placebo (*n* = 116) [60]. Although no significant differences were noted in both groups with regard to efficacy, SAMe was found to be safe and well tolerated, with predominantly mild GI side effects, including nausea, vomiting, abdominal discomfort, abdominal pain, diarrhea, constipation, and dyspepsia. The risk of GI discomfort may be more pronounced at higher doses of SAMe (3200 mg/day), as reported by Sakurai et al. in a randomized clinical trial [61]. A 6-week double-blind placebo-controlled trial demonstrated SAMe as a safe and well-tolerated agent up to a daily dose of 1600 mg [62]. No significant differences were reported in the rate of side effects between SAMe (1600 mg/day) and placebo groups. Moreover, no manic or psychotic symptoms were exhibited in SAMe-treated patients.

### 2.4. The Possible Influence of New Dosage Forms

SAMe is reported to be labile, as it degrades rapidly after oral administration, and several patents for stable salts of SAMe have been granted, including formulations with p-toluene sulfonate (tosylate) and 1,4-butanedisulfonate salts [63]. A limited number of clinical studies have shown low bioavailability of SAMe after oral intake, though the supplement is readily absorbed from the GI tract, which indicates a significant first-pass metabolism in the liver [64]. Initial approaches utilized intravenous preparations of SAMe with commercially stabilized tosylate, but these were generally limited to a short duration of a few weeks. With the increased availability of enteric coated forms, formulated with stable salts, such as SAMe-tosylate or SAMe-1,4-butanedisulfonate, clinical trials were able to extend them to much longer periods of several months [63].

The bioavailability of a novel enteric coated oral formulation of SAMe (i.e., MSI-195 (SAMe disulfate tosylate) was evaluated by Cameron et al. in healthy subjects using a cross-over study design [65]. MSI-195 was administered in different doses to healthy subjects, including both males and females (400, 800, and 1600 mg/day), and the results indicated that the MSI-195 formulation was well tolerated with a similar adverse event profile to that of a conventionally available SAMe formulation (SAMe Complete™). The relative bioavailability of MSI-195 was 2.8-fold higher than that of SAMe Complete™ based on area-under-curve (AUC) calculations. Moreover, food possessed a significant effect on the absorption of LSI-195, as the time to reach maximum absorption (T_max_) was 13 h in a fed state, compared with 4.5 h in a fasting state, and AUC was reduced to 55% of that of the fasting state. Francioso et al. (2021) demonstrated the improved chemical stability and pharmacokinetic parameters of a novel formulation of SAMe (phytate salt) in rats [66]. SAMe phytate was administered orally to rats, and the results were compared with SAMe tosylate, with the phytate showing significantly better pharmacokinetic parameters. Phytate anions offer better protection to the SAMe molecule, preventing degradation perhaps due to steric hindrance provided by the counterion.

## 3. Gastrointestinal Microbiota and Neurobiological Pathways Related to Depression

A great number of microorganisms, including bacteria, viruses, fungi, protozoans, and archaea, are known to colonize the human body, living in coexistence with their host, and these are referred to as microbiota or normal flora [67]. Joshua Lederberg was the first to introduce the concept of a human microbiota to the scientific community, who defined it as “the ecological community of symbiotic and pathogenic microorganisms that literally share our body space and have been all but ignored as determinants of health and disease” [68]. Bacterial species are the most extensively studied microorganism for their beneficial role in the human microbiome, while other species are not particularly known for their health effects in relation to their host. More importantly, microbiota colonize every part of the human body exposed to the external environment, with the GI tract being the most heavily colonized, with the colon alone containing about 70% of all microbes present in the body [67,69,70].

The Firmicutes and Bacteroidetes phyla are the most common examples of gut microbiota, representing approximately 90% of microbial species present in the GI tract. Other examples of gut microbiota include the phyla of Actinobacteria, Fusobacteria, Proteobacteria, and Verrucomicrobia [71]. The most essential functions of gut microbiota are regulation of digestion, production of vitamins (B12 and K), restriction of the growth and/or activity of nasty pathogens, production of short-chain fatty acids (SCFAs), and metabolism of essential substances, such as bile acids, sterols, and drugs [72]. Gut microbiota are divided in two groups, that is, beneficial flora and opportunistic bacteria. The first group are also known as gut housekeepers, containing species of *Lactobacteria*, *Bifidobacterium*, *Enterococci*, *Propionobacteria*, and *Peptostreptococci*. The second group can harm the body if it gets an opportunity, comprising *Bacilli*, *Bacteriodes*, *Clostridia*, *Actinobacteria*, *Enterobacteria*, *Peptococci*, *Streptococci*, *Staphylococci*, and *Saccharomyces* [73]. An imbalance between the species of these two groups results in gut dysbiosis, providing a road map to the pathogenesis of chronic disorders, including GI inflammatory disorders, colorectal cancer, metabolic disorders, autoimmune diseases, neurodegenerative disorders, and psychiatric issues [73,74,75].

The interaction between the gut and the brain was first recognized by physicians centuries ago [76]. The correlation between altered bowel function and depression was realized in the 16th century, and this was scientifically corroborated by Manning et al. in 1978 by linking irritable bowel syndrome (IBS) with psychological stress, with some authors reporting about 50% of patients with comorbid anxiety or depression [76,77]. The pathways and mechanisms by which the gut microbiome, gut, and brain function are interlinked are collectively referred to as the microbiome–gut–brain axis, a concept described by the American scientist Gershon in the 20th century [78,79]. Gut microbial alteration could increase the intestinal permeability, upregulate the systemic inflammation, regulate the monoamine neurotransmitter release, and alter the function of the hypothalamic–pituitary–adrenal (HPA) axis. Stress is also a known factor to increase the intestinal permeability, providing an opportunity to bacteria to translocate across the intestinal mucosa, thus accessing neuronal cells of the enteric nervous system [80]. The benefits of gut microbial modulation on decreasing inflammatory tone via the reduction of intestinal permeability have been reported widely in the literature. Pretreatment of rats with *Lactobacillus farciminis* reduced the intestinal permeability and prevented associated HPA hyperactivity [81]. The gut microbiota communicated with the brain via immunoregulation, endocrine, and neuronal regulation pathways [82].

As far as the immunoregulation pathway is concerned, the gut microbiome regulates the inflammatory processes in the CNS by modifying the function of lymphocytes and the production of cytokines [83,84,85]. The endocrine regulation pathway is described by the release of glucocorticoids, mineralocorticoids, and catecholamines from the HPA axis when confronted with stress or other stimulants, resulting in the alteration of the composition of the gut microbiota, followed by enhanced gut epithelium permeability and immune responses [81,86,87]. The involvement of the neuronal regulation pathway is demonstrated by the role of the gut microbiota in the secretion and regulation of neurotransmitters (norepinephrine, serotonin, gamma-aminobutyric acid (GABA), or dopamine) and the release of cytokines from stimulated intestinal lymphocytes [79,88].

The vagus nerve forms a direct connection between the gut and the brain, and this may transmit neuronal, hormonal, and bacterial changes in the bowel directly to the brain [89]. Moreover, by the secretion of neurotrophins and proteins, for example, BDNF, synaptophysin, and postsynaptic density (PSD)-95, the gut microbiome affects brain development and plasticity [90,91]. Depressive disorders are associated with alterations in the HPA axis and immune responses [92]. A rodent model of depression showed a decrease in the diversity of the gut microbiota, with a hyperactive HPA axis-upregulated expression of proinflammatory cytokines [93,94]. Aizawa et al. reported a decrease in *Bifidobacterium* and *Lactobacillus* in patients with MDD [95], while Jiang et al. noted an increase in Bacteroidetes, Proteobacteria, and Actinobacteria with a decrease in Firmicutes in MDD [96]. Numerous strategies have been proposed to prevent or treat neuropsychiatric disorders through the regulation of imbalances in the composition of gut microbiota, including the use of probiotics [97].

### 3.1. Probiotics and Antidepressant Potential

Probiotics are live microorganisms that confer health benefits when supplemented in sufficient amounts via improvement or restoration of the gut flora. They can be consumed as food supplements or in functional foods and generally include species of *Bifidobacterium*, *Lactobacillus*, *Saccharomyces*, some strains of *Escherichia coli*, and some Gram-positive cocci [98]. Probiotic species with healthy benefits in psychiatric patients are referred to as psychobiotics, acting by producing and delivering neuroactive substances that may act on the gut–brain axis and, in some cases, may serve as antidepressants [76]. Growing evidence suggests that probiotics have gained significant attention due to their wide array of clinical uses, ranging from applications in GI tract disorders to those in extraintestinal diseases [99]. Multiple studies have explored the link between probiotic species and mood disorders, considering the role of the gut–brain axis in the pathophysiology of depressive disorders [100]. Table 2 summarizes the clinical trials, illustrating the effects of SAMe in depressive disorders.

In a randomized double-blind placebo-controlled study, 44 adult patients with IBS, diarrhea or mixed stool pattern, and concomitant mild to moderate anxiety and depression based on the Hospital Anxiety and Depression Scale (HADS) were treated with placebo or *Bifidobacterium longum* NCC3001 for 6 weeks [101]. The IBS symptoms, level of anxiety and depression, and quality of life of the patients were evaluated at weeks 0, 6, and 10. In addition, brain activation patterns, urine metabolome profiles, fecal microbiota, serum markers of inflammation, and neurotrophin and neurotransmitter levels were analyzed. At week 6, depression scores were significantly reduced in *B. longum*-treated patients, with improvements in quality of life, while anxiety and IBS parameters did not change in either group. Functional magnetic resonance imaging (fMRI) showed a decreased response to negative emotional stimuli in the amygdala and fronto-limbic regions in the *B. longum* group. Moreover the *B. longum* group showed decreased urine levels of methylamines and aromatic amino acids, but no difference was found for either group in fecal microbiota profiles, neurotrophin and neurotransmitter levels, and inflammatory markers.

Probiotic supplementation in patients with MDD resulted in an improvement of Beck Depression Inventory (BDI) scores, as compared with placebo, in a randomized clinical trial [102]. A total of 110 patients were randomized to receive probiotic supplement (*Lactobacillus helveticus* R0052 and *B. longum* R0175), prebiotic (galacto-oligosaccharide), or placebo for 8 weeks. A total of 81 patients completed the study. Probiotic supplementation resulted in significant decreases in BDI scores, increases in tryptophan/isoleucine ratio, and reductions in kynurenine/tryptophan ratio, compared with placebo. No such effects were seen with prebiotics. Kato-Kataoka et al. (2016) supplemented healthy medical students with fermented milk containing *Lactobacillus casei* strain Shirota (*n* = 24) or placebo (*n* = 23) for 8 weeks for the prevention of the onset of physical symptoms [103]. The physical symptoms (common abdominal and cold symptoms) and the total number of days during which the students had these symptoms were significantly lower in the *L. casei*-treated group. *L. casei* significantly increased serotonin levels; however, no difference was found across both groups with regard to HADS-anxiety, HADS-depression, Self-Rating Depression Scale (SDS), and Pittsburgh Sleep Quality Index (PSQI) scores.

Steenbergen et al. (2015) demonstrated the effects of a multispecies probiotic supplement (*Bifidobacterium lactis* W52, *B. bifidum* W23, *Lactobacillus acidophilus* W37, *L. brevis* W63, *L. casei* W56, *L. salivarius* W24, and *L. lactis* W19, and *L. lactis* W58) in the amelioration of cognitive reactivity to sad mood [104]. The participants were supplemented with a multispecies probiotic supplement or placebo for 4 weeks. Assessment of the Leiden Index of Depression Sensitivity Scale-Revised (LEIDS-r) showed a considerable reduction in cognitive reactivity to depression, in particular, rumination and aggressive thoughts, in probiotic-supplement-treated participants for the postintervention period when compared with the preintervention period. A randomized placebo-controlled double-blind trial conducted by Benton et al. indicated an improvement of mood with the consumption of a probiotic-containing yoghurt in those individuals whose mood was initially poor [105]. A total of 132 healthy individuals (mean age: 61.8 years) were voluntarily included in the study and were randomized to receive either yoghurt containing *L. casei* Shirota (10^8^/mL) or placebo for 3 weeks. A total of 124 candidates finished the study with results taken at baseline and after 10 and 20 days. Mood was determined using the Profile of Mood States (POMS) scale, and cognition was assessed using the Wechsler Memory Scale.

Kazemi et al. (2019) conducted a double-blind placebo-controlled randomized clinical trial, recruiting 110 patients with MDD, aged 18–50 years, to evaluate the effects of probiotic (*L. helveticus* R0052 and *B. longum* R0175) and prebiotic (galacto-oligosaccharide) supplementation on circulatory proinflammatory cytokines and urinary cortisol levels [106]. The patients were treated with prebiotic, probiotic, or placebo for 8 weeks. No change was observed on the level of inflammatory markers with these supplements, but probiotic supplementation yielded a significant decrease in urinary cortisol levels and BDI scores. Probiotic administration possessed beneficial effects on depression and metabolic parameters in patients with MDD, as reflected by a randomized double-blind placebo-controlled trial [107]. Forty patients within the age range of 20–55 years were treated with probiotic supplement containing *L. acidophilus* (2 × 10^9^ CFU/g), *L. casei* (2 × 10^9^ CFU/g), and *B. bifidum* (2 × 10^9^ CFU/g), or placebo for 8 weeks. Significant improvements in BDI scores, serum insulin concentration, homeostasis model assessment of insulin resistance (HOMA-IR) and high-sensitivity C-reactive protein (hs-CRP), and glutathione concentrations were seen in patients with probiotic administration.

A randomized triple-blind placebo-controlled trial showed an improvement of cognitive symptoms in patients supplemented with Ecologic^®^ Barrier (2.5 × 10^9^ CFU/g) for 8 weeks compared with a placebo group especially in mild to moderate depression [108]. The formulation (Ecologic^®^ Barrier) contained *B. bifidum* W23, *B. lactis* W51, *B. lactis* W52, *L. acidophilus* W37, *L. brevis* W63, *L. casei* W56, *L. salivarius* W24, *L. lactis* W19, and *L. lactis* W58 (total cell count: 1 × 10^10^ CFU/day), and the subjects were provided with two sachets of the formulation daily. However, no significant alteration in the gut microbiota of depressed individuals was observed with the intervention. The dosage size of probiotic supplementation might not be sufficient to detect any changes with the gut microbial diversity, as observed previously by Saxelin et al. (1995) [109]. Similarly, multistrain probiotic supplementation in combination with biotin showed an overall beneficial effect on the clinical treatment of inpatients diagnosed with MDD in a randomized double-blind controlled trial [110]. The subjects enrolled received probiotic strains plus biotin or placebo plus biotin for 4 weeks, where both groups showed improvement of psychiatric symptoms at the end of the treatment period, and no significant differences were recorded between the intervention and placebo groups. Different reasons may be responsible, such as short duration of treatment, small sample size, inpatient settings, and difference at the baseline in the smoking status of patients. The main changes observed in the intervention group were abundance of *Ruminococcus gauvreauii* and *Coprococcus 3* strains and elevation of inflammatory regulatory and metabolic pathways.

Nevertheless, these preliminary studies showed promising results and offer a road map for future research and clinical studies to build upon. In clinical practice, probiotics may be useful as adjunct agents to potentiate the effects of cognitive behavioral therapy and pharmacological treatments. However, the literature should be expanded before consideration of this versatile class of nutraceuticals as part of the regular treatment of mild to moderate depression. The dosages and timeframes of specific probiotic supplementation should be defined, and additional analytical methods should be employed to investigate specific gut microbiota strains and strategies on how to maximize the antidepressant effects of these probiotic supplementations.

**Table 2:** Antidepressant efficacy of probiotic supplementation.

Mental Condition	Study Design	Intervention	Main Findings	References
IBS with mild to moderate depression and anxiety	Double-blind RCT. A total of 44 adult patients (age: 26–58 years) with IBS and mild to moderate depression and anxiety were selected.	*B. longum* (1 × 10^10^ CFU/g powder) (*n* = 22) or placebo (*n* = 22) for 6 weeks.	*B. longum* reduced depression scores but not anxiety scores with improvements in quality of life.	[101]
MDD	Double-blind RCT. A total of 110 patients were randomized to receive probiotic, prebiotic, or placebo.	Probiotic supplement (*L. helveticus* R0052 and *B. longum* R0175), prebiotic (galacto-oligosaccharide), or placebo for 8 weeks.	Probiotic supplementation decreased BDI scores, increased tryptophan/isoleucine ratio, and reduced kynurenine/tryptophan ratio, compared with placebo. No such effects were seen with prebiotics.	[102]
Healthy medical students	Double-blind placebo-controlled trial. Healthy medical students were recruited in the trial to evaluate the effects of probiotic species in the prevention of the onset of physical symptoms.	Fermented milk containing *L. casei* strain Shirota (*n* = 24) or placebo (*n* = 23) for 8 weeks.	Fermented milk reduced the onset of physical symptoms, with a significant elevation of fecal serotonin levels. No difference was found in HADS-anxiety, HADS-depression, SDS, and PSQI scores in both groups.	[103]
Healthy individuals	Triple-blind placebo-controlled randomized pre- and postintervention assessment design. A total of 40 healthy individuals participated in the evaluation of the effects of a multispecies probiotic supplement in the reduction of cognitive reactivity to sad mood.	Multispecies probiotic supplement (*B. lactis* W52, *B. bifidum* W23, *L. acidophilus* W37, *L. brevis* W63, *L. casei* W56, *L. salivarius* W24, *L. lactis* W19, and *L. lactis* W58) or placebo for 4 weeks.	LEIDS-r showed a significant reduction in the cognitive reactivity to depression, in particular, rumination and aggressive thoughts, in the probiotic-supplement-treated group.	[104]
Depressed mood	Double-blind randomized placebo-controlled trial. A total of 132 healthy individuals (mean age: 61.8 years) with poor mood voluntarily participated in the study.	Yoghurt containing *L. casei* Shirota (10^8^/mL) or placebo for 3 weeks.	Improvement of mood with the consumption of a probiotic-containing yoghurt.	[105]
MDD	Double-blind placebo-controlled randomized clinical trial. A total of 110 patients with MDD (age: 18–50 years) were recruited in the trial.	Probiotic (*L. helveticus* R0052 and *B. longum* R0175), prebiotic (galacto-oligosaccharide), or placebo for 8 weeks.	No change in the level of inflammatory markers. Probiotic supplementation significantly decreased urinary cortisol levels and BDI scores. No antidepressant effects were seen with prebiotics.	[106]
MDD	Double-blind placebo-controlled randomized clinical trial. A total of 40 patients with MDD and in the age range of 20–55 years were recruited in the study.	Probiotic supplement containing *L. acidophilus* (2 × 10^9^ CFU/g), *L. casei* (2 × 10^9^ CFU/g), and *B. bifidum* (2 × 10^9^ CFU/g) or placebo for 8 weeks.	Significant improvement in BDI scores, serum insulin concentration, HOMA-IR and hs-CRP, and glutathione concentrations with probiotic administration.	[107]
Depressive symptoms in mild to severe depression	Triple-blind placebo-controlled randomized clinical trial. A total of 71 patients with mild to severe depression were randomized in the intervention (mean age: 36.65 years) or placebo (mean age: 35.49 years) groups.	Ecologic^®^ Barrier (2.5 × 10^9^ CFU/g) containing *B. bifidum* W23, *B. lactis* W51, *B. lactis* W52, *L. acidophilus* W37, *L. brevis* W63, *L. casei* W56, *L. salivarius* W24, *L. lactis* W19, and *L. lactis* W58 (total cell count: 1 × 10^10^ CFU/day) or placebo for 8 weeks.	Significant improvement of cognitive symptoms in the intervention group as compared with the placebo group, especially in patients with mild to moderate depression. No significant effect on gut microbial alteration.	[108]
Inpatients diagnosed with depression	Randomized double-blind controlled trial. A total of 82 inpatients diagnosed with depression were randomized in the intervention group (*n* = 42) or placebo (*n* = 40).	OMNi-BiOTiC^®^ Stress Repair (*B. bifidum* W23, *B. lactis* W51, *B. lactis* W52, *L. acidophilus* W22, *L. casei* W56, *L. paracasei* W20, *L. plantarum* W62, *L. salivarius* W24, and *L. lactis* W19) plus 125 mg D-biotin or placebo plus D-biotin for 4 weeks.	No significant differences between both groups in psychiatric symptoms. More abundance of *Ruminococcus gauvreauii* and *Coprococcus 3* strains and elevation of inflammatory regulatory and metabolic pathways in the intervention group.	[110]

### 3.2. Mechanistic Targets

The anti-inflammatory, antioxidant, and immune-regulatory properties; the downregulation of the HPA axis, and increased biosynthesis of neurotransmitters are essential mechanisms exerted by probiotics that offer the hope of confronting the underlying causes of depression, with the aim of long-term remission of disease [111,112,113,114,115]. The antioxidant and anti-inflammatory actions of probiotics may result in increasing BDNF levels [115]. *Komagataella pastoris* KM71H pretreatment showed a prevention against depression in mice via the restoration of mRNA levels of nuclear factor kappa B, interferon γ, interleukin 1β, and indoleamine 2,3-dioxygenase; attenuation of oxidative stress in the prefrontal cortex, hippocampus, and intestines; and reduction of corticosterone levels [116]. Probiotic bacterial strains are reported to increase the secretion of neurotransmitters, such as GABA (*L. casei* and *L. rhamnosus*), serotonin (*L. helveticus*), norepinephrine (*L. helveticus*), and histamine (*L. reuteri*) [117]. Histamine secretion could reduce the release of proinflammatory cytokines by intestinal epithelial cells, which may decrease circulatory inflammatory markers (LPS, IL-6, and corticosterone) and ultimately result in the prevention of inflammation-induced decreases in hippocampal BDNF [117]. *B. infantis* has been found to significantly increase the plasma concentration of the serotonergic precursor tryptophan in rats, with a decrease in inflammatory markers, including interferon (IFN-γ), TNF-α, and IL-6 [114]. Probiotics influence SCFA production, where SCFAs may modulate the synthesis of several neurotransmitters associated with cognition and behavior. In particular, propionic and butyric acids upregulate the expression of tryptophan and tyrosine hydroxylase, the enzymes that are involved in the synthesis of noradrenaline, serotonin, and dopamine [118].

*L. rhamnosus* was observed to have effects on neurotransmission in an in vivo study using a mouse model system. This bacterial strain was found to exert selective regulation of GABA expression in the brain, in conjunction with the reduction of depression-like behavior. Conversely, vagotomized mice did not show the same effects, leading to the conclusion that the vagus nerve might play an important role in gut-mediated effects on the brain [119]. These results were supported by a study that investigated direct vagus nerve stimulation (VNS) as an effective treatment strategy for treatment-resistant depression, reporting improvement in patients with mild to moderate depression [120]. *L. plantarum* may increase butyrate and butyrate-producing bacteria (*Lactobacillus*, *Bacteroidetes,* and *Roseburia*), which help in strengthening the intestinal barrier, regulating BDNF expression, and reducing inflammation in the brain [121,122,123]. *L. plantarum* may also increase dopamine levels in the prefrontal cortex to prevent overactivation of the HPA axis [124]. *B. infantis* upregulates the tryptophan hydroxylase-1 activity of enterochromaffin cells through butyrate production, resulting in enhanced circulating serotonin levels and a strengthened intestinal barrier, lowering indoleamine 2,3-dioxygenase activity and increasing circulating tryptophan, both of which affect the central serotonin system and BDNF expression [125,126].

*L. paracasei* PS23 reversed corticosterone-induced depressive behaviors in mice [127]. Both *L. paracasei* PS23 and fluoxetine were shown to reverse low BDNF levels induced by corticosterone in the hippocampus and serotonin and dopamine levels in the hippocampus and prefrontal cortex. A mouse model of depression demonstrated attenuation of anxiety-like behaviors induced by chronic mild stress, an increase in *Lactobacillus* abundance, and the reversal of stress-induced immune changes in the hippocampus by affecting IFN- γ, TNF-α, and indoleamine 2,3-dioxygenase-1 levels with the supplementation of a multistrain probiotics treatment (*L. helveticus* R0052, *L. plantarum* R1012, and *B. longum* R0175), which suggests that the gut microbiota–inflammation–brain axis is a possible target for alleviating anxiety and depression [128]. Bioactive components secreted by probiotic species may also help in improving depressed moods; that is, polysaccharides secreted by *B. infantis* may decrease circulating IL-6 levels affecting the central norepinephrine system; H_2_O_2_ secreted by *L. reuteri* decreases indoleamine 2,3-dioxygenase activity and circulating kynurenine and diacylglycerol kinase, inhibiting proinflammatory pathways; and exopolysaccharides secreted by *L. kefiranofaciens* possess immunomodulatory activities, preventing HPA axis overactivation [117].

Moreover, numerous controversial points are still to be addressed, including leaky gut syndrome and BBB permeability in relation to depressed mental states, as their association has already been explained through neuropsychiatric disorders [129].

## 4. The Possible Combination of Probiotics and S-Adenosyl Methionine

Adjuvant administration of SAMe with SSRIs in the dose range of 800–1600 mg/day to patients with resistant depression could result in remission from complex resistant depressive states in about 36% of patients; however, the remaining 64% of patients may have a suboptimal response to the augmentation therapy [42]. Interestingly, resistant depression is usually accompanied by systemic inflammation induced by gut dysbiosis, and the imbalance in neurotransmitter production may be a direct result of this gut dysbiosis [130,131]. Gut microbiota also play a role in the first-pass metabolism of natural products, including SAMe, through the alteration of host pathways for metabolism and transport. Dysbiosis of the gut microbiome may directly affect the therapeutic efficacy of these products [132,133]. Lactic acid bacteria (LAB) have been evaluated for their SAMe-producing ability, and researchers found that *B. bifidum* BGN4 produced a significantly higher amount of SAMe when compared with other strains of *Bifidobacterium* or *Lactobacillus* [134].

Thus, concomitant administration of probiotics could be an effective treatment strategy in patients with depressed mood, particularly in resistant cases, as it can ameliorate dysbiosis, which may possibly result in the attenuation of systemic inflammatory processes and the improvement of the therapeutic efficacy of supplements such as SAMe. On the other hand, SAMe reduces inflammatory mediators through a reduction in proinflammatory bacteria in the gut, increasing glutathione levels and positively regulating growth factor signaling, which may also improve the therapeutic response of pharmacological agents and natural supplements in these patients [131]. A clinical trial, with reference number NCT03932474, demonstrated a fast and clinically significant improvement of mild to moderate symptoms with the supplementation of SAMe and *L. plantarum* HEAL9 in adult patients with SD [135]. In a double-blind placebo-controlled study, 90 subjects aged 18–60 years with mild to moderate depression, according to the International Classification of Diseases (ICD-10) diagnostic criteria, were randomized to receive a combination of SAMe (200 mg) and *L. plantarum* HEAL9 (1 × 10^9^ CFU) (*n* = 46) or placebo (*n* = 44) for 6 weeks. The results showed a considerable improvement of symptoms of depression, anxiety, cognitive and somatic components after 2 weeks of treatment in patients supplemented with the combination of SAMe and *L. plantarum* HEAL9.

## 5. Concluding Remarks

SAMe possesses significant mood-enhancing potential owing to its multiple mechanistic targets, particularly transmethylation pathways and monoamine neurotransmitters. Its antidepressant efficacy has been evidenced in numerous clinical studies, whether used alone to combat mild to moderate depressive symptoms or in combination with traditional antidepressant medications in moderate to severe instances of the disease. Furthermore, oral dosage of SAMe is readily safe up to 1600 mg, with no serious adverse concerns. Unlike other antidepressant drugs, SAMe is not associated with sexual and cognitive/memory dysfunction. However, caution should be exercised with the use of SAMe in bipolar patients due to the risk of hypomanic or manic symptoms. In the light of available literature data, it is best to use SAMe in patients with mild to moderate depression, rather than relying on conventional antidepressant medicines, which may carry an increased risk of toxicity in such patients.

Despite having antidepressant efficacy, suboptimal responses have been observed with SAMe in the majority of cases, especially with resistant depression, where systemic inflammation and imbalances in neurotransmitter production induced by gut dysbiosis are central parts of the pathophysiological mechanism. Probiotic species are designated as health-promoting agents and are essential in counterbalancing gut dysbiosis, which may be associated with several intestinal and extraintestinal disorders. In depression, probiotic supplementation may counter inflammatory and immune-mediated signaling and HPA axis overactivation, with upregulated BDNF expression and increased production of neurotransmitters (GABA, serotonin, norepinephrine, and dopamine). Certain probiotic species have been proven in clinical studies to have considerable antidepressant efficacy. The concomitant supplementation of SAMe and probiotics could be an effective strategy in the remission of depressive symptoms, as this combination would address gut dysbiosis with attenuation of systemic inflammatory responses and improvement in the healing efficacy of SAMe. Formulation of SAMe using new techniques, such as oral disintegrated tablets, could increase its bioavailability, which may decrease the daily dose requirement of SAMe, reducing the possibility of side effects associated with higher dosages, such as GI upsets.

As far as future perspectives are concerned, several studies have shown that SAMe undergoes extensive hepatic first-pass metabolism after oral intake, reducing its therapeutic efficacy. Several formulations of SAMe with stable salts in the form of enteric coated tablets have been designed to counter the issues of low bioavailability. It has been suggested that SAMe and probiotic strains be formulated together using novel formulation techniques, with the aim of increased bioavailability of SAMe and their synergistic antidepressant effects. As much as available studies are concerned regarding SAMe and probiotics supplementation in mild to moderate depression, most of the studies are carried out in patients with MDD and based on which the clinical efficacy of SAMe and probiotic strains cannot be reflected in mild to moderate depression. Further clinical trials are needed to confirm the clinical effectiveness of both supplements individually in subclinical and mild to moderate depression. Moreover, clinical studies are needed to confirm the efficacy and safety of SAMe and probiotic strains together in novel formulations in subjects with mild to moderate depression.
